# Supplementary material for: Option talk and risk communication with people with limited health literacy: A qualitative focus group study with key stakeholders
Source: PLoS One. 2025 Aug 29;20(8):e0330191. doi: 10.1371/journal.pone.0330191 (PMC12396664; doi:10.1371/journal.pone.0330191)
Supplement: S1 Appendix — (PDF) [file pone.0330191.s001.pdf]

S1 Appendix 1 - PtDA examples

CONSULT KAART

ARTROSE IN DE KNIE:

behandelmogelijkheden

Heeft u last van artrose in uw knie? Deze Consultkaart kan u en uw orthopeed helpen om uw mogelijkheden voor behandeling te bespreken. Let op: een kunstknie wordt vaak pas later gekozen, als de andere behandelingen niet of onvoldoende werken. Hoeveel klachten u heeft kan erg veranderen. Soms kan een halve knieprothese of een standcorrectie ook mogelijk zijn. Deze behandelingen worden in deze Consultkaart niet besproken. Vraag eventueel uw orthopeed hiernaar.

BEHANDEL-MOGELIJKHEDEN	UW LEEFSTIJL VERANDEREN	PIJNSTILLERS SLIKKEN	INJECTIES IN UW KNIE	U KRIJGT EEN KUNSTKNIE *(knieprothese)*
**Hoe werkt de behandeling?**	- U krijgt adviezen voor het veranderen van uw leefstijl.<br>- Het advies kan gaan over actief bewegen en voeding.<br>- Een fysiotherapeut of diëtist kan u eventueel begeleiden.<br>- U kunt daarnaast eventueel pijnstillers slikken.	- U slikt paracetamol.<br>- Helpt de paracetamol niet? Dan kunt u een NSAID slikken. Dit is een pijnstiller en een ontstekingsremmer die u op recept kunt krijgen.<br>- Heeft u veel bijwerkingen van de NSAID? Dan kunt u minder NSAID slikken én paracetamol.<br>- Helpt de NSAID ook niet? Dan kunt u Tramadol slikken. Dit is een zwaardere pijnstiller die u op recept kunt krijgen.<br>- Het kan helpen als u daarnaast uw leefstijl verandert.	- Uw arts geeft u een injectie met Corticosteroïd in uw knie.<br>- Het kan helpen als u daarnaast uw leefstijl verandert.<br>- Een injectie met Hyaluronzuur, bloedplaatjes (PRP) of stamcellen raden wij niet aan. We weten niet goed wat het effect is.	***In het ziekenhuis***<br>- U gaat onder narcose of krijgt een ruggenprik.<br>- Uw arts vervangt uw knie door een kunstknie.<br>- U blijft 2 tot 5 dagen in het ziekenhuis.<br><br>***Als u weer thuis bent***<br>- U gebruikt 3 tot 6 weken bloedverdunners.<br>- U loopt 4 tot 6 weken met krukken.<br>- U krijgt 3 tot 6 maanden fysiotherapie.<br>- Na ongeveer een half jaar loopt u weer normaal.<br>- Het totale herstel kan een jaar duren.
**Heb ik minder pijn na de behandeling?**	Door een sterker lichaam en betere conditie heeft u waarschijnlijk na een paar weken minder pijn.	Vaak vermindert de pijn direct. Werkt een pijnstiller niet? Dan kunt u een sterkere pijnstiller proberen.	Meestal verminderen de zwelling en de pijn binnen 1 week. De injectie werkt meestal meerdere weken tot maanden. Als de klachten terugkomen, kan de arts u eventueel opnieuw een injectie geven.	Meestal wordt de pijn na de operatie geleidelijk minder. Soms wordt de pijn niet minder. Na een jaar zeggen 93 van de 100 patiënten (93%) dat de pijn minder of veel minder is geworden.
**Wat zal ik na de behandeling weer kunnen?**	Als de pijn minder wordt, kunt u zich makkelijker bewegen. Door beweging kan de pijn óók weer minder worden.	Als de pijn minder wordt, kunt u zich makkelijker bewegen. Door beweging kan de pijn óók weer minder worden. Gaat u intensief bewegen? Dan kan het helpen als u vooraf pijnstillers slikt.	Als de pijn minder wordt, kunt u zich makkelijker bewegen. Door beweging kan de pijn óók weer minder worden.	De meeste patiënten kunnen zich makkelijker bewegen.<br>- Rustige sporten zoals wandelen, zwemmen, golfen en fietsen kunt u veilig doen.<br>- Balsporten, zoals basketbal, voetbal en volleybal, of hardlopen kunt u beter niet doen.
**Wat zijn risico's en mogelijke bijwerkingen van de behandeling?**	Er zijn geen risico's of bijwerkingen. Als u meer beweegt en afvalt, wordt de kans dat de artrose erger wordt juist kleiner.	- Paracetamol heeft weinig bijwerkingen.<br>- NSAID's zorgen bij 10 tot 30 van de 100 patiënten (10-30%) voor maag-darmklachten, zoals misselijkheid, buikpijn en diarree. Soms zorgt NSAID voor stoornissen in de nierfunctie, hoge bloeddruk of huiduitslag.<br>- Tramadol zorgt bij meer dan 10 van de 100 patiënten (>10%) voor misselijkheid en duizeligheid. 1 tot 10 van de 100 patiënten (1-10%) heeft last van obstipatie, braken, een droge mond, zweten, hoofdpijn, slaperigheid, vermoeidheid en verwijde bloedvaten.	- Na veel injecties is er een klein risico dat het kraakbeen in uw knie beschadigt. Hierdoor wordt de artrose erger.<br>- Heel soms krijgt een patiënt een allergische reactie of een infectie.<br>- Ongeveer 2 van de 100 patiënten (2%) hebben een paar dagen na de injectie gewrichtspijn en zwelling.<br>- Bij patiënten met diabetes kunnen de bloedsuikers tijdelijk ontregeld zijn.	- Minder dan 1 van de 100 patiënten (1%) krijgt een infectie aan de wond die behandeld moet worden.<br>- Heel soms krijgt een patiënt een infectie aan de kunstknie. Dan is een nieuwe operatie nodig.<br>- Soms laat de kunstknie na een tijdje los. Dan is ook een nieuwe operatie nodig.<br>- 2 van de 100 patiënten (2%) krijgen bloedstolsels in het been.<br>- De risico's op complicaties zijn groter als u andere aandoeningen heeft, rookt of te zwaar bent.<br>- De kans dat de kunstknie vervangen moet worden, hangt af van uw leeftijd en hoeveel u beweegt. Over het algemeen moet een kunstknie na 15 tot 20 jaar vervangen worden.

De Consultkaart Artrose in de knie is ontwikkeld met ondersteuning van het Kennisinstituut van Medisch Specialisten.
Voor meer informatie zie www.consultkaart.nl.

Patiëntenfederatie Nederland samen de zorg beter maken
NEDERLANDSE ORTHOPAEDISCHE VERENIGING | NOV
2016

PATIENT+ CZ

ᴀ A A Inloggen Startpagina

*This is a part of the PtDA (website)

Informatie | Vergelijken | Belangrijke punten | Uw voorkeur | Afsluiting

Informatie
- Wat is artrose?
- De behandelingen

Geen operatie
- De resultaten

Een operatie
- Herstel na de operatie
- De resultaten
- De complicaties

Welke behandelingen zijn er?

Knie artrose kan op twee manieren worden behandeld:

- **Geen operatie**
- **Een operatie**

LET OP: Een arts zal niet meteen gaan opereren. De arts zal pas een operatie voorstellen als andere behandelingen niet meer werken.

Vragen? | Aantekeningen | Vorige | Volgende

Overlijden

Heel soms overlijdt iemand tijdens de operatie of kort daarna. Dit gebeurt bijna nooit, namelijk bij minder dan 1 op de 100 mensen.[6]

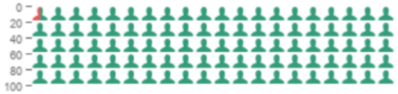

Nog een operatie

5 van de 100 mensen moeten binnen 10 jaar opnieuw geopereerd worden. Bijvoorbeeld omdat de prothese los zit of omdat er iets anders mis is.

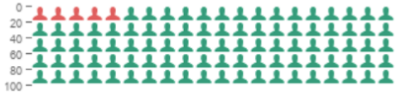

Vergelijk de behandelingen

	Geen operatie	Operatie
**Wat betekent het?**	Regelmatig bewegen.<br>Spieren sterker maken.<br>Afvallen.<br>Pijnstillers slikken.	Operatie waarbij u een knieprothese krijgt.<br>U bent in slaap of uw been is verdoofd.<br>1 tot 3 dagen in het ziekenhuis.<br>4 tot 6 weken met een hulpmiddel lopen.<br>3 tot 6 maanden fysiotherapie.<br>Volledig herstel kan een jaar duren.
**Bij hoeveel mensen helpt het?**	Fysiotherapie en pijnstillers helpt bij 52 van de 100 mensen tegen de pijn.	93 van de 100 mensen hebben minder pijn en kunnen meer bewegen.
**Wat zijn de complicaties en nadelen ?**	Er zijn geen complicaties of bijwerkingen van meer bewegen en afvallen.<br>Paracetamol: Bijna geen bijwerkingen bij normaal gebruik.<br>Ibuprofen of naproxen: maagklachten. 20 van de 100 mensen worden misselijk of krijgen buikpijn van de pijnstillers. Bij lang gebruik meer kans op maagbloedingen.<br>Cortico-steroïd injecties: allergische reactie of verhoging van de bloedsuikerspiegel bij diabetes.	Niet goed kunnen buigen van de knie.<br>Wondontsteking bij 2 van de 100 mensen.<br>Trombose (bloedprop) bij 2 van de 100 mensen. Om dit te voorkomen neemt u een bloedverdunnend medicijn.<br>17 van de 100 mensen hebben na de operatie nog steeds pijn.<br>Bij 5 van de 100 laat de prothese los.<br>Bij 18 van de 100 mensen moet de prothese na 25 jaar vervangen worden.<br>Als u een hartziekte of longziekte heeft, zijn de risico's voor u groter. Ook als u rookt of overgewicht heeft loopt u meer gevaar bij een operatie.
**Hoe lang duurt het voordat er resultaten te verwachten zijn?**	Door sterkere spieren en meer bewegen na een paar weken minder pijn.<br>Pijnstillers helpen snel. Vaak al na een uur of anders na 1 of 2 dagen.<br>Corticosteroïd injecties geven meestal binnen een week resultaat. Na enkele weken tot maanden kunnen de klachten terugkomen.	De pijnklachten door de operatie, worden na een tijdje minder.<br>De meeste mensen lopen na 6 weken zonder hulpmiddelen.<br>Volledig herstel kan een jaar duren.
**Wanneer adviseert de arts deze behandeling?**	Als u voor het eerst klachten heeft.<br>Afvallen is belangrijk voor mensen met overgewicht.	Als andere behandelingen geen effect hebben.

CONSULTKAART IN BEELD (PHAROS)

**This is a part of the PtDA (pdf)*

CONSULT KAART

OVERZICHT KEUZES

Behandelingen bij Artrose in de Knie

**Als het kraakbeen in de knie verandert, kan dit zorgen voor een stijf gevoel en pijn.
Dit heet artrose in de knie.**

**Er zijn verschillende behandelingen mogelijk.
Deze kaart laat 5 verschillende behandelingen zien.
De arts praat met u over de verschillende behandelingen.
Samen met de arts maakt u een keuze welke behandeling het beste bij u past of mogelijk is.
De kaart laat zien wat de behandeling is, hoe lang de behandeling is en welke gevolgen dit heeft voor u.**

A KEUZE: Uw manier van leven veranderen

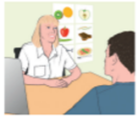

☐

B KEUZE: Fysiotherapie/oefentherapie

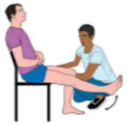

☐

C KEUZE: Pijnstillers

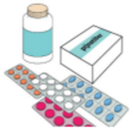

☐

D KEUZE: Prik in uw knie

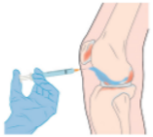

☐

E KEUZE: Een kunstknie

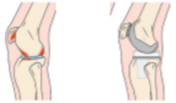

VOOR operatie NA operatie

☐

meer informatie: www.reumazorgnederland.nl

ReumaZorg Nederland — Nederlandse Orthopaedische Vereniging | NOV

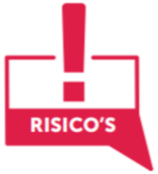

4 Dit zijn de risico's:

U kunt heel soms last krijgen van:
- Een allergische reactie.
- Een infectie in de knie.
- Pijn en een dikke knie (een paar dagen na de prik).
- Heeft u suikerziekte (diabetes)? Uw bloedsuiker kan de eerste paar dagen na de prik te hoog of te laag zijn.

- Na veel prikken kan het kraakbeen in uw knie verder beschadigen. Hierdoor wordt de artrose erger.

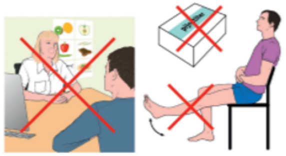

5 Voor wie is deze behandeling?

U kunt deze behandeling volgen als:
- Afvallen niet helpt.
- Oefeningen niet helpen.
- Pijnstillers niet genoeg helpen en u pijn blijft houden.
