# Supplementary material for: Option talk and risk communication with people with limited health literacy: A qualitative focus group study with key stakeholders
Source: PLoS One. 2025 Aug 29;20(8):e0330191. doi: 10.1371/journal.pone.0330191 (PMC12396664; doi:10.1371/journal.pone.0330191)

S2 Appendix 2 - Examples used in focus groups with people with LHL

Consultkaart in beeld (PHAROS) available online: https://www.pharos.nl/kennisbank/keuzekaart-in-beeld-artrose-in-de-knie/

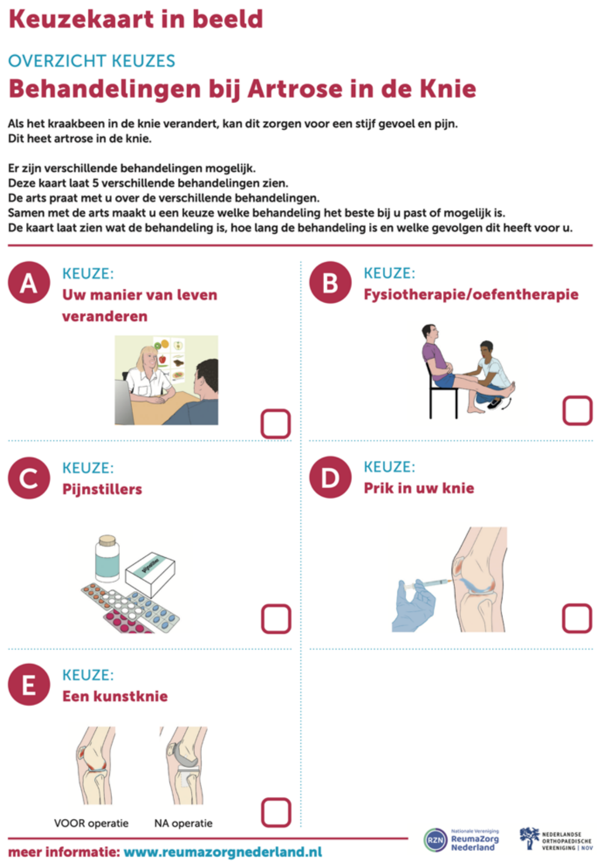

Keuzekaart in beeld

OVERZICHT KEUZES

Behandelingen bij Artrose in de Knie

Als het kraakbeen in de knie verandert, kan dit zorgen voor een stijf gevoel en pijn.
Dit heet artrose in de knie.

Er zijn verschillende behandelingen mogelijk.
Deze kaart laat 5 verschillende behandelingen zien.
De arts praat met u over de verschillende behandelingen.
Samen met de arts maakt u een keuze welke behandeling het beste bij u past of mogelijk is.
De kaart laat zien wat de behandeling is, hoe lang de behandeling is en welke gevolgen dit heeft voor u.

A KEUZE: **Uw manier van leven veranderen**

B KEUZE: **Fysiotherapie/oefentherapie**

C KEUZE: **Pijnstillers**

D KEUZE: **Prik in uw knie**

E KEUZE: **Een kunstknie**

VOOR operatie NA operatie

meer informatie: www.reumazorgnederland.nl

Nationale Vereniging ReumaZorg Nederland

Nederlandse Orthopaedische Vereniging | NOV

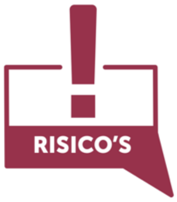

4 Dit zijn de risico's:

Er is een kleine kans op:
- Bloedpropjes in uw bloed (trombose).
- Een infectie in de wond.
- Een infectie in de kunstknie.
- De kunstknie blijft niet goed op z'n plek zitten en laat los.

- Deze risico's worden groter als u rookt, te zwaar bent of andere ziekten heeft.

- Soms moet de kunstknie binnen 15 jaar weer vervangen worden, als de klachten erger worden.

Numerical risk communication – natural frequency

4 Dit zijn de risico's:

Bloedprop (Trombose)

De eerste weken na de operatie heeft u een verhoogde kans op een bloedprop. Daarom krijgt u een bloedverdunnend medicijn. Toch krijgen **2 van de 100 mensen trombose**, meestal binnen 3 maanden na de operatie.

Visual risk communication - icon array

4 Dit zijn de risico's:

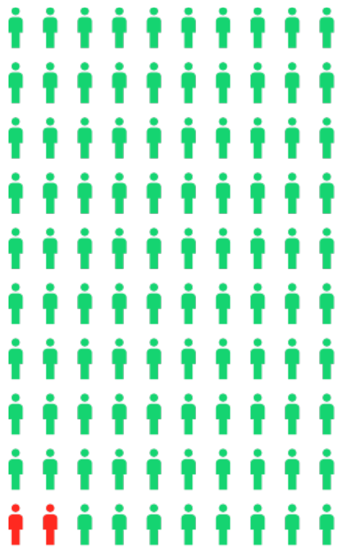

Visual risk communication – bar chart

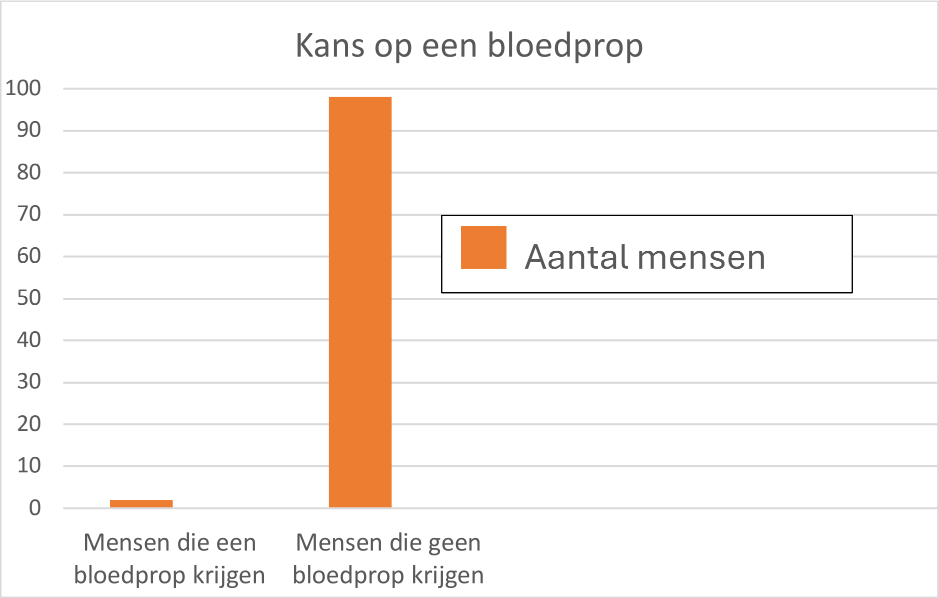

Supplement: S2 Appendix — (PDF) [file pone.0330191.s002.pdf]
