# Supplementary material for: Option talk and risk communication with people with limited health literacy: A qualitative focus group study with key stakeholders
Source: PLoS One. 2025 Aug 29;20(8):e0330191. doi: 10.1371/journal.pone.0330191 (PMC12396664; doi:10.1371/journal.pone.0330191)
Supplement: Attachment — Submitted filename: Response to Reviewers.docx [file pone.0330191.s003.docx]

# Response letter Ref.: PONE-D-24-06379; Title: " Option talk and risk communication in shared decision-making for people with limited health literacy - a focus group study with experts, doctors, and people with limited health literacy.”

30 October 2024

*Steve Zimmerman, PhD, Senior Editor, PLOS ONE*

Dear *Steve Zimmerman*,

**RE: Major revision PONE-D-24-06379; Title: "Option talk and risk communication in shared decision-making for people with limited health literacy - a focus group study with experts, doctors, and people with limited health literacy.”**

Thank you for giving us the opportunity to revise and resubmit our manuscript to *PLOS ONE.*

We have provided a point-by-point response to reviewers and have specified where these changes appear in the manuscript. We used Track Changes to mark any changes made in the manuscript and uploaded a manuscript version with tracked changes, a clean manuscript version and the rebuttal letter. Please see below for our responses to the reviewer comments.

In response to the editor’s comments, we ensured that our manuscript meets PLOS ONE's style requirements, including those for file naming and added more information concerning the Data Availability Statement.

Sincerely,

Romy Richter, on behalf of all authors

Reviewers' comments:

## Reviewer #1:

**Reviewer comment:**

The study presents a topical issue, and its research is important if we want to apply, in a generalized way, less paternalistic and more participatory health models, where patients can make their own decisions. On the other hand, the application of qualitative methods helps to elicit patients' preferences and, in many cases, can offer results closer to the study objective than quantitative ones.
However, the difficulty in the design and the tools to be used on the target population are key elements. In this regard, from my point of view, the paper should explain in more detail the following points:
a) The choice of knee arthritis PtDA as an example.
It is deduced from reading the paper that the knee arthritis PtDA was used only as an example. Would it have been possible to work with several tools from different diseases with more/less aggressive treatments to analyze the possible consequences of SDM in different circumstances?
Undoubtedly, SDM will be more or less complex depending on the disease and its treatments, and using a specific example in the Focus Groups can overestimate or undervalue some results.
It is also important to consider that the target population of this work may be affected by social inequalities that affect, above all, preventive health measures. Adding an example related to these measures could be an alternative for a more concise understanding of patient preferences. Do the authors believe that the discussion and results would have been very different in this case?
This topic could be incorporated into the Discussion section.

**Response to comment**

Thank you for your constructive comment. The choice of the knee arthritis patient decision aid (PtDA) was based on a small sample of newly developed PtDAs by the Dutch Centre of Expertise on Health Disparities (Pharos). At the time of our study, there were only four available PtDAs specifically designed for individuals with limited health literacy, and the knee arthritis PtDA was the only one that incorporated risk communication – specifically verbal risk communication. The other PtDAs did not include risk communication, which made the knee arthritis example particularly suitable for our analysis. We agree that SDM will be more or less complex depending on the disease and its treatments. We therefore regard knee arthritis a good example, as it is a condition that is easily understood by patients, as many can relate to or imagine the experience of knee pain. This helped us engage participants effectively during focus group discussions.

We selected this PtDA not because of the specific disease, but because of its unique approach to presenting information on treatment options and their benefits and harms to patients with limited health literacy. Our focus was on the communication strategies, not on the condition itself, allowing us to generate general recommendations for improving shared decision making (SDM) processes. Moreover, our work builds on previous research, including a comprehensive systematic review of risk communication strategies for people with limited health literacy, and an analysis of Dutch PtDAs, which has informed our approach. Therefore, we are confident that our findings are not overly influenced by the specific choice of the knee arthritis PtDA, but rather reflect broader, applicable insights.

Incorporating more examples from different diseases, including those involving preventive health measures, might indeed have provided additional insights. However, we believe the communication strategies we analyzed are broadly applicable across different health contexts. Our goal was not to demonstrate the specific consequences of shared decision making (SDM) in particular diseases, but rather to offer insights and recommendations on effective communication strategies, especially for option talk and risk communication. We appreciate your suggestion and will consider it for future work. Additionally, we will incorporate your comment into the Discussion limitation section to acknowledge the potential influence of disease context on SDM outcomes.

We agree that social inequalities can significantly impact preventive health measures. However, we believe that the communication strategies we examined, particularly for option talk and risk communication, are broadly applicable across different contexts, including preventive health. While the specific example used may influence certain nuances in patient responses, we do not expect the overall recommendations on communication strategies to change significantly. Exploring preventive health measures in future research would enrich understanding of shared decision making in these populations. However, this represents a different scope and would be more suited for a separate project.

**Added text in discussion – strengths and limitations, see page 25:**

Our focus groups used a single PtDA for knee arthritis, which may limit the generalizability of our findings to other conditions. While exploring PtDAs from different diseases could provide broader insights, our goal was not to examine disease-specific SDM outcomes but to offer recommendations on effective communication strategies, particularly for option talk and risk communication.

**Reviewer comment:**
b) Table 2 should be revised. So, for example:
1. The column with the number of participants in each GF does not correspond to the number cited in the abstract, nor does the number of rows correspond to the number of participants cited in the table heading.
2. We don't know which 2 experts dropped out of the study. Did this abandonment change the number of participants, or was it already considered at the time of preparing the table?
3. The number of people participating in the focus groups is low in relation to the usual average. The reason for this choice should be justified.
4. Participants in the expert and patient groups were subdivided into two FGs, although there were 11 and 6 people, respectively (an acceptable number for a single group). Explain why.

**Response to comment:**

1. We have revised the sentence in the abstract and added a legend to Table 2 to ensure the number of participants in each focus group (FG) aligns with the numbers cited in the abstract and the table instructions.

**Methods:** Five focus groups (FGs) were conducted with a) experts on health literacy, SDM, or risk communication (RC**) (two FGs, n=5 and n=6);** b) experienced doctors (one FG, n=8), and c) people with LHL (two FGs, 2x n=3).

**Table legend, see page 11:** *Focus groups with experts and people with LHL were divided into two groups. Grey shading indicates these divisions

2. The two experts who dropped out were accounted for when preparing the table. Therefore, the number of participants listed in Table 2 reflects the final number after these dropouts.
3. We acknowledge that the number of participants in some of the focus groups is lower than the typical average. This is specifically the case for the focus group with people with limited health literacy. We purposefully decided to keep the size of these FGs small, based on recommendations from the Dutch Centre of Expertise on Health Disparities (PHAROS) with expertise in working with people with limited health literacy. They advised smaller group sizes to foster more open discussion and richer data collection, which is particularly important when working with this population. Due to logistical constraints and experts' availability, we conducted two focus groups for experts. This ensured each expert could participate meaningfully without time conflicts. We believe that the other focus groups have a reasonable group size as shown in methodology literature. Meaningful discussions were reached also in the focus groups with less participants. As shown in methodology literature smaller focus groups work better when sensitive topics are discussed or when the participants have considerable expertise in or experience of the topic (Doody 2012).

- Plummer P. Focus group methodology. Part 1: Design considerations. Int J Ther Rehabil. 2017 Jul 2;24(7):297–301. Available from: <https://www.magonlinelibrary.com/doi/abs/10.12968/ijtr.2017.24.7.297>
- Doody O, Slevin E, Taggart L. Focus group interviews in nursing research: Part 1. British Journal of Nursing. 2013 Jan 9;22(1):16–9. Available from: <https://pubmed.ncbi.nlm.nih.gov/23299206/>
- Morgan D. Focus Groups as Qualitative Research. Focus Groups as Qualitative Research. SAGE Publications, Inc.; 2012. Available from: /record/1997-97533-000

1. The decision to subdivide the expert and patient groups into two focus groups was deliberate. For the experts, this approach was also due to practical reasons and logistical constraints. Given the full agenda and the availability of the experts, finding a single time slot that could accommodate everyone proved challenging. By creating two groups, we ensured that each expert could fully participate in a setting that allowed for meaningful contributions, without the risk of time constraints or scheduling conflicts. Additionally, we followed best practices, which suggest that focus groups should ideally have no more than six participants to encourage dynamic discussions. This approach was also applied to the focus group with people with limited health literacy to ensure deeper engagement and to create a comfortable environment for all participants to share their views.

We hope these explanations address your concerns and provide clarity regarding the design choices made for the focus groups.

**Reviewer comment:**
The facilitators and barriers of the health system organization are not reflected in any case in the paper. Only, on one occasion, the possible lack of time of the professionals is mentioned. The literature has repeatedly cited the importance of these elements faced by professionals when implementing the SDM. In addition, the specific needs of the target population mean that these external variables, related to the healthcare context, may have more relevance than in a study with another target population.
This topic could be addressed in the Discussion section.

**Response to comment:**

Thank you for your comment. Our primary focus was on developing recommendations for communication strategies, specifically regarding option talk and risk communication, rather than on examining broader facilitators and barriers to shared decision making (SDM). While we acknowledge the importance of systemic factors such as healthcare organization and time constraints, these were beyond the scope of our study. Our goal was to provide insights on improving communication within SDM, assuming that space and time are available for such consultations. However, we recognize the potential relevance of these external factors, particularly for our target population, and will address this briefly in the Discussion section.

**Text in discussion, see page 25:**

Broader systemic factors, related to the healthcare organization, such as time constraints, were not the focus of our study. While these are well-known challenges in implementing SDM, addressing them was beyond our scope. Given the specific needs of our target population, these systemic barriers could play a more significant role in real-world SDM processes, requiring further exploration in future research.

## Reviewer #2:

**Reviewer comment:**

Dear editor Thank you very much for choosing me as a referee. The article with the title "Belililil" is a very important and popular topic in the world. Although the article is well written, it is recommended that the following points be considered:
The purpose is clearly stated and relevant. It addresses an important issue in healthcare, focusing on shared decision-making (SDM) and risk communication (RC) for people with limited health literacy (LHL).

**Response to comment:**

Thank you for your encouraging comment.

**Reviewer comment:**
1. The methodology is well-defined, using focus groups (FGs) with different stakeholders. However, the sample size for people with LHL (2x n=3) is quite small, which might limit the generalizability of the findings.

**Response to comment:**

We acknowledge that the number of participants in some of the focus groups is lower than the typical average. This it's the case for the focus group with people with limited health literacy that decision was based on recommendations from the Dutch Centre of Expertise on Health Disparities (PHAROS) with expertise in working with people with limited health literacy. They advised smaller group sizes to foster more open discussion and richer data collection, which is particularly important when working with this population. However, we believe that the other focus groups have a reasonable group size as shown in methodology literature such:

- Plummer P. Focus group methodology. Part 1: Design considerations. Int J Ther Rehabil [Internet]. 2017 Jul 2 [cited 2020 Aug 11];24(7):297–301. Available from: <https://www.magonlinelibrary.com/doi/abs/10.12968/ijtr.2017.24.7.297>
- Doody O, Slevin E, Taggart L. Focus group interviews in nursing research: Part 1. British Journal of Nursing [Internet]. 2013 Jan 9 [cited 2020 Aug 11];22(1):16–9. Available from: <https://pubmed.ncbi.nlm.nih.gov/23299206/>
- Morgan D. Focus Groups as Qualitative Research [Internet]. Focus Groups as Qualitative Research. SAGE Publications, Inc.; 2012 [cited 2020 Aug 11]. Available from: /record/1997-97533-000

The decision to subdivide the expert and patient groups into two focus groups was deliberate. For the expert group, we followed best practices, which suggest that focus groups should ideally have no more than six participants to encourage dynamic discussions. This approach was also applied to the focus group with people with limited health literacy to ensure deeper engagement and to create a comfortable environment for all participants to share their views.

**Reviewer comment:**
2. The use of both inductive and deductive content analysis is appropriate for qualitative research.

**Response to comment:**

Thank you very much for your feedback.

**Reviewer comment:**
3. The results are comprehensive and highlight key points such as the importance of tailoring SDM to the patient’s context and the challenges of identifying patients with LHL.

**Response to comment:**

Thank you very much for your feedback.

**Reviewer comment:**
4. The variation in views on the level of detail required for effective RC is well-documented, showing the complexity of the issue.

**Response to comment:**

Thank you very much for your feedback.

**Reviewer comment:**
5. The conclusion effectively summarizes the findings and suggests that the strategies identified for people with LHL may be relevant to a broader patient population.

**Response to comment:**

Thank you very much for your feedback.

**Reviewer comment:**
6. The call for further research is appropriate, given the small sample size and the exploratory nature of the study.

**Response to comment:**

Thank you very much for your feedback.

**Reviewer comment:**
7. The article is generally clear and well-structured. However, some sentences are quite long and could be broken down for better readability.

8. For example, the sentence “FGs included a brief presentation defining SDM, option talk, and RC and introducing different RC strategies and decision aids for guided group discussions” could be split into two sentences for clarity.

**Response to comment:**

Thank you very much for your valuable feedback. We have carefully reviewed the manuscript again, making adjustments to improve the phrasing and sentence structure where possible. See adjusted sentences from manuscript text below. As we split the sentence you suggested into two, we consequently needed a few additional words compared to the original abstract.

**Manuscript text:**

See page 2: FGs included a brief presentation defining SDM, option talk, and RC. Different RC strategies and decision aids were introduced to support guided group discussions.

See page 3: ~~SDM is a process in which the healthcare provider and patient discuss which medical management plan is best for the patient, considering all relevant options (option talk) with their benefits and harms and the patient´s preferences and context.~~ 🡪 SDM is a process in which the healthcare provider and patient discuss which medical management plan is best for the patient. This involves considering all relevant options (option talk) with their benefits and harms and the patient´s preferences and context (1).

See page 4: ~~A clear and understandable presentation of all medical options, the so-called option talk in SDM and RC, is essential for all patients as it leads to a transparent basis for decision-making through presenting evidence on the benefits and harms of options~~ 🡪 A clear and understandable presentation of all medical options, known as option talk in SDM and RC, is essential for all patients. It provides a transparent basis for decision-making by presenting evidence on the benefits and harms of each option.

See page 5: ~~Consequently, we chose the epistemological orientation of subjectivism, referring to many interpretations of reality and participants’ RC strategies being subjectively developed concerning the clinical situation.~~ 🡪 Consequently, we chose the epistemological orientation of subjectivism, which acknowledges multiple interpretations of reality. Since participants’ RC strategies are subjectively developed in relation to the clinical situation (21).

See page 7: ~~At the same time, an inductive approach was applied to capture and categorise new aspects related to views on option talk and RC strategies without preconceived ideas, including challenges, dilemmas, and factors associated with content and context.~~ 🡪 At the same time, an inductive approach was applied to capture and categorise new aspects related to views on option talk and RC strategies without preconceived ideas. This included identifying challenges, dilemmas, and factors associated with content and context (29).

See page 8: ~~The richness of data was enhanced by including experts and GPs with diverse backgrounds, and the representativeness of people with LHL was encouraged by assigning recruitment to an expertise Centre for health literacy.~~ 🡪 The richness of data was enhanced by including experts and GPs with diverse backgrounds. The representativeness of people with LHL was encouraged by assigning recruitment to an expertise Centre for health literacy.

See page 25: ~~It could be considered to integrate the exploration of the patient’s level of knowledge and their context (step 0) more structurally into the process of SDM to help tailor option talk and RC and make numbers more meaningful.~~ 🡪 It could be considered to integrate the exploration of the patient’s level of knowledge and their context (step 0) more structurally into the process of SDM. This could help tailor option talk and RC to make numbers more meaningful to patients.

**Reviewer comment:**
9. There are minor grammatical errors, such as “patient´s context” which should be “patient’s context.”

**Response to comment:**

Thank you for pointing out that aspect. We adjusted it to “patient’s context”.

**Reviewer comment:**
10. Ensure consistent use of abbreviations. For instance, “RC” is introduced without explanation in the “Methods” section.

**Response to comment:**

Thank you for your comment. We have reviewed the use of abbreviations. We can confirm that the abbreviation "RC" for risk communication was introduced in both the abstract and the introduction. For readability, we also introduced the abbreviation in the Methods section again.

**Reviewer comment:**
11. The flow of the article is logical, moving from purpose to methods, results, and conclusion.

**Response to comment:**

Thank you very much for your positive feedback on the clarity and logical flow of our study.

**Reviewer comment:**
12. Ensure that each section transitions smoothly to the next. For example, the transition from the “Methods” to the “Results” section could be improved by adding a brief summary sentence.

**Response to comment:**

Thank you for your suggestion. While we appreciate the idea, we are not entirely certain what is meant by 'a brief summary sentence'. Given the length of the article, we believe it is important to maintain a clear and concise structure to keep the content focused and ensure readability.

**Reviewer comment:**
13. Ensure all statements are backed by appropriate references. If any claims or findings are based on previous research, cite them accordingly.

**Response to comment:**

Thank you very much for your feedback. We carefully reviewed the document again, ensuring that references are included where necessary.

**Reviewer comment:**
14. Ensure consistent formatting throughout the document, including headings, subheadings, and bullet points.

**Response to comment:**

Thank you for your comment. We have reviewed the document again to ensure that the formatting is correct.

**Reviewer comment:**
15. Use bullet points or numbered lists where appropriate to enhance readability, especially in the “Results” section.

**Response to comment:**

Thank you very much for your constructive comment. We carefully reviewed the Results section. We have adjusted the first paragraph and the second part “characteristics of participants”, merging them into one section under the heading “Focus Groups and participant characteristics.” This section is now divided into subheadings for the focus groups with experts, general practitioners, and individuals with limited health literacy, with Table 2 providing detailed information on the focus groups and participants.

To enhance clarity in the main findings, we adjusted the bullet point formatting. As in the previous version, each theme is introduced in a dedicated section for better readability. For sections containing participant quotes, we believe bullet points or numbering would not be appropriate. After careful consideration, we made these adjustments and decided to keep the rest of the Results section unchanged to preserve its clarity and flow.

**Manuscript text, see page 9 and 10:**

**FGs and participant characteristics**

We conducted five FGs. Table 2 provides further information on the FGs and participants. All participants attended the FGs on time, and the atmosphere was open and welcoming. There were no cancellations for the FGs with experts and individuals with limited health literacy (LHL), although two participants withdrew from the GP FG due to personal reasons. There were no negative power dynamics in any of the FGs.

**FG experts**

In both FGs with experts, there was a balanced representation of SDM and health literacy. Most of the input gathered in the first FG with experts was also mentioned in the second FG with experts. Only a few new themes emerged. Many of the experts were familiar with both each other and the moderator. In the expert FGs, more females than males participated.

**FGs GPs**

In the FG with GPs, most participants from the Dutch College of General Practitioners (DCGP) knew each other, and the moderator (TvdW) already knew three of the participants. Although we have no indication that this influenced the FG, we cannot completely exclude some influence. In the FG with GPs, one participant spoke a larger amount. There seemed to be much respect for this person's seniority and work experience. Hence, during the GP FGs, not all participants spoke equally. The moderator tried to stimulate participation by explicitly targeting the quieter participants.

**FGs people with LHL**

The gender ratio for the FGs with people with LHL was balanced. People with LHL obtained at least primary education except for one person. Five participants with LHL were Dutch; one had a migration background. One person had paid work.
