# Supplementary material for: Option talk and risk communication with people with limited health literacy: A qualitative focus group study with key stakeholders
Source: PLoS One. 2025 Aug 29;20(8):e0330191. doi: 10.1371/journal.pone.0330191 (PMC12396664; doi:10.1371/journal.pone.0330191)
Supplement: Attachment — Submitted filename: Response letter PONE-D-24-06379R1] .docx [file pone.0330191.s004.docx]

Authors response to Reviewer's comments

**Reviewer comment:**
Title:
- Title too long, remove: a focus group study with experts, doctors, and people with limited health literacy.
- Include the type of methodology.

**Response to comment**

Thank you for your comment. We adjusted the title to: "Option talk and risk communication with people with limited health literacy: a qualitative focus group study with key stakeholders.”

**Reviewer comment:**
**Abstract:**
- Make the objective shorter and more direct.

**Response to comment**

Thank you for your comment. We adjusted the objective to: To explore stakeholders´ views on acceptable and feasible strategies for discussion of treatment options and risk communication with people with limited health literacy (LHL in the context of shared decision-making (SDM)).

**Reviewer comment:**
**Abstract:**
- The number of participants is a result (what was done) and not a method (how it was designed to include and/or exclude, select and/or calculate the sample)

- The methodology needs to have more information about the type of study, data collection method, sample selection method, when and where data collection took place, and data analysis method.
- Conclusion must respond to the objective of the study.

**Response to comment**

Thank you for your valuable feedback. We have revised the abstract to better distinguish between methodology and results, ensuring that the number of participants is appropriately presented as a result. Additionally, we have expanded the methodology section to specify the study type and sampling strategy. Given the abstract's length constraints and the need to avoid information overload, we have focused on these key aspects. The data collection method (focus groups) and analysis approach (inductive and deductive content analysis) were already included. Further details on sample selection criteria, data collection timeframe, and location are provided in the methods section of the manuscript. Lastly, we have refined the conclusion to more directly address the study’s objective.

**New abstract:**

**Purpose:** To explore stakeholders´ views on acceptable and feasible strategies for discussion of treatment options and risk communication with people with limited health literacy (LHL in the context of shared decision-making (SDM)).

**Methods:** This qualitative descriptive study used purposive sampling to conduct focus groups with stakeholders including experts in health literacy, SDM, or risk communication (RC); experienced General Practitioners (GPs); and individuals with LHL. Each session included a brief presentation defining SDM, option talk, and RC, followed by an introduction to various RC strategies and decision aids to facilitate discussion. Verbatim transcripts were analysed **by two independent researchers** using **inductive and deductive content analysis.**

**Results: Five focus groups were conducted, involving experts** (two FGs, n=5 and n=6)**, GPs** (one FG, n=8) and people with LHL (two FGs, 2x n=3)**. Experts and GPs emphasised the need to tailor communication to the** patient’s **context and noted challenges in identifying patients with LHL. All participants highlighted the importance of using illustrations of treatment options (e.g. knee injections) to support the discussion of options. Views on the level of detail required for RC varied, with some GPs questioning whether RC was understandable to people with LHL. Most people with LHL preferred RC in natural frequencies and icon arrays but noted that RC can fan fear. GPs found contextualisation (e.g. comparing the probability of a treatment outcome with the probability of a car accident) a helpful strategy, but patients found it confusing. Decision aids were seen as supportive for RC.** Overall, people with LHL preferred their doctor to discuss options face-face with them, using a layered step-by-step manner, adding details on RC as preferred by patients.

**Conclusion:** SDM for people with LHL benefits from a tailored, layered approach with visual aids. These strategies are potentially useful for all patients, but further research is needed to confirm this.

**Introduction:**
- Too long may be more limited to the purpose of the work.
- The purpose of the abstract and introduction are not standardized.

**Response to comment**

Thank you for your feedback. We have shortened the introduction and checked for alignment with the purpose of the study. The adjusted introduction can be found in the main document.

**Abstract:**

To explore stakeholders´ views on acceptable and feasible strategies for discussion of treatment options and risk communication with people with limited health literacy (LHL in the context of shared decision-making (SDM)).

**Introduction:**

To gain an understanding of acceptable and feasible strategies to support SDM in practice, we aim to explore views and preferences regarding the discussion of treatment options (option talk) and RC among (a) experts in SDM, health literacy and RC, (b) experienced GPs, and (c) people with LHL.

**Methods:**
- Having the estimated number of eligible people and how many were selected could improve understanding of the study.
- It talks about the recruitment period, but not about when data collection took place.
- How many interviewers? Was there prior training?

**Response to comment**

Thank you for your feedback. We have added information on the data collection period in the methods section and provided further details on the interviewers under the "FG Interviews" subsection. Additionally, we have clarified the number of interviewers involved and whether prior training was conducted. Details on participant selection and recruitment were also added under *Participant Selection and FG intervies* (please see details under reviewer comment “results”)

**FG interviews**

Due to the COVID-19 pandemic, FGs with experts and GPs took place online via the video platform Zoom©**, all but two of the GPS who were approached participated in the study**. **The FGs with experts and GPs were conducted in January and February 2022.** The FG with people with LHL took place at PHAROS **in May 2022.** The FGs were conducted in Dutch and lasted one and a half hours, with a break between the two main topics. FGs were audio and/or video recorded, and field notes were taken during the FGs with experts and GPs. We started with the two expert FGs, followed by the FG with the GPs and ended with the two FGs with people with LHL. **A different interviewer was assigned to each participant group. The focus groups with individuals with LHL were conducted by an experienced interviewer from PHAROS. Focus groups with experts were led by co-author JR, a professor in health literacy, while those with GPs were conducted by co-author TvdW, a professor in shared decision-making. Both co-authors have extensive experience in conducting focus groups.**

**Results:**
- How many were recruited? How many accepted? How many participated?

- If the research was carried out through a focus group, that is, through observation of the phenomenon through a group, why were the results presented individually?

**Response to comment**

Thank you for your feedback. We clarified those recruitment aspects. There were two dropout in the focus group with doctors due to personal reasons. Therefore, the number of participants presented in Table 2, plus the two dropouts in the doctor group, reflects the total number of individuals recruited.

**Manuscript text:**

*Participant Selection*

We used purposive sampling. Eleven experts and 30 GPs were contacted by email through the research team and their network from 01/10/2021 to 31/01/2022. To recruit GPs, names and contact details of potential participants were gathered in consultation with project advisors and with a working group on diversity and international health from the Dutch College of GPs (DCGP). All the experts and ten GPs responded positively to participating. Six people with LHL were recruited by The Dutch Centre of Expertise on Health Disparities (PHAROS) between February and May 2022. All participants had to be proficient in the Dutch language. Inclusion criteria can be found in Table 1.

*FG interviews*

Due to the COVID-19 pandemic, FGs with experts and GPs took place online via the video platform Zoom©, all but two of the GPs who were approached participated in the study.

Regarding the presentation of results, while focus groups capture group discussions, our analysis also considered individual perspectives within those discussions to provide a more comprehensive and nuanced understanding of the data. This approach highlights diverse viewpoints while still respecting the group context.

**Manuscript text:**

Data analyses: Although data were collected through focus groups, the presentation of results also attends to individual perspectives expressed within the group discussions. This approach enables a more comprehensive and nuanced understanding of the data by highlighting diverse viewpoints while preserving the group context.
